# Micromechanical Modeling for Tensile Properties of Wood Plastic Composites: Use of Pruned Waste from Pecan Orchards as Sustainable Material for Reinforcement of Thermoplastic Composite

**DOI:** 10.3390/polym14030504

**Published:** 2022-01-27

**Authors:** Juan Miguel Díaz-Mendoza, Delia J. Valles-Rosales, Young H. Park, Ronald C. Sabo

**Affiliations:** 1 Department of Industrial and Manufacturing Engineering, Universidad Autónoma de Ciudad Juarez, Av. Del Charro 450, Juarez Chih 32410, Mexico; 2 Department of Industrial Engineering, New Mexico State University, 1060 Frenger Mall Dr. MSC 4230, Room 294, Las Cruces, NM 88003, USA; 3 Department of Mechanical Engineering, New Mexico State University, 1780 E. University Ave, Las Cruces, NM 88003, USA; ypark@nmsu.edu; 4 USDA Forest Products Laboratory, One Gifford Pinchot Drive, Madison, WI 53726, USA; Ronald.sabo@usda.gov

**Keywords:** tensile strength, sustainable, wood plastic composites, pecan waste, particle size, micromechanical modeling

## Abstract

Wood plastic composites (WPCs) specimens containing high-density polyethylene (HDPE) and wood pruning waste were manufactured and evaluated for their mechanical properties. Pecan waste was used as an accessible and sustainable source in this study, and the effects of its particle size and concentration on WPC strengths were evaluated. Pecan waste was milled and sieved to various particle sizes, and testing samples were fabricated by mixing them in a twin-screw extruder and injection molding. A coupling agent was used to create a stable bond between the HDPE and wood. Both tensile modulus and strength were increased with an increasing pecan flour concentration up to about 60 weigh percent. A micromechanical model is proposed for predicting the mechanical properties of the wood flour/fiber reinforce composite. This model uses a correction factor of an elliptical of carried sizes and shapes. The preliminary results of the model have a high correlation with the experimental values of the composite in all mesh sizes.

## 1. Introduction

The use of composites containing natural fibers is growing rapidly around the world. The combination of thermoplastic polymers (e.g., polyolefins) with natural fibers has been used to develop wood plastic composites (WPCs) widely. WPCs have been developed from a variety of agricultural and forest resources, including wood flour and other types of fibers from plants [1,2]. In particular, the use of agricultural waste fibers has reached a production of 140 billion metric tons annually around the globe [3]. A literature review [1,4] shows an increased use of agro-waste as a reinforcement option in plastic composites. Many of these composites are based on the use of waste from crops after harvesting. Some of these natural materials are obtained from orchard pruning residues. Currently, this waste is typically either burned or mixed in the soil as fertilizer. Using an agro-waste approach can create composites that would be greener and less harmful to the environment. In the manufacturing industry, there is an increase in environmental awareness and government regulations forcing companies to look for environmentally friendly materials that can be biodegradable, ecologically friendly, low cost with good physical properties and energy-efficient [5]. Natural fibers, including agricultural waste, are considered to be a viable option to meet the manufacturing industry requirements [6]. Different waste sources have been studied to characterize WPCs. For example, Fernandez-Garcia et al. [7] analyzed the mechanical properties of palm tree pruning in particleboard. In the case study from Merida, Mexico [8], Cruz-Estrada et al. studied the use of pruning waste from trees and recycled HDPE [8]. In another study, conducted by Pinzón, Moreno and Saron [9], the mechanical properties of low-density polyethylene with waste pinewood were analyzed. An analysis by Valles-Rosales et al. [10] reviewed the potential use of chili stalks waste as a fiber for polypropylene. In a study presented by Oliver-Ortega et al., waste from rapeseed and polypropylene was used to fabricate specimens, which were tested for mechanical properties for potential construction material [11]. Sutivisedsak et al. [12] developed WPCs from three different nutshell flours such as almond, pistachio and walnut waste. Jorda-Reolid et al. [13] analyzed the waste of argan nutshell with bio-based polyethylene. These composites have properties to potentially be used in several areas such as building, construction, automotive and furniture. Even so, the physical requirements on each application are different; the composites have a wide variety of applications. Therefore, an evaluation of the properties of WPCs with various materials is important.

**Figure 1:**
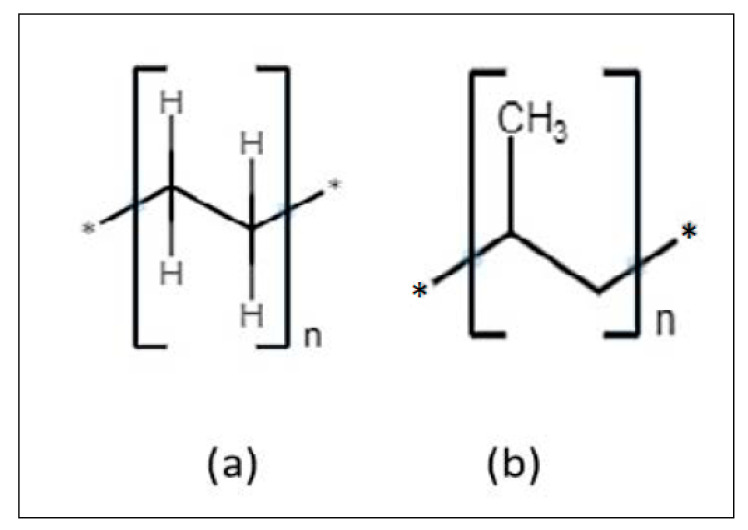
(**a**) Polyethylene, (**b**) Polypropylene [17].

Various studies have focused on the thermal, rheological and mechanical properties of WPCs [11]. In particular, efforts have been devoted to obtaining good mechanical properties for new WPCs [13]. For example, Maldas et al. [14] discussed the effect of additives on fiber dispersion and mechanical properties in high-density polyethylene and peanut hull and a 10% pecan shell particle composite. The study was conducted to analyze the effect of maleate polyethylene (MAPE) and peroxide on mechanical characteristics. The results indicated an improvement of fiber dispersion when MAPE was used. The mechanical properties of tensile strength were significantly improved when maleate polyethylene was used; however, the tensile modulus remained unchanged. In a study conducted by Sutivisedsak et al. [12], WPCs were created from three different nutshell flours such as almond, pistachio and walnut. The results obtained showed lower mechanical properties than the base polymer matrix (polypropylene and poly lactic acid). In another study, Adhikary, Pang and Staiger [15] investigated WPCs based on recycled and virgin HDPE with wood flour, and they observed a significant an increase in tensile strength. Some previous studies have also analyzed the effect of wood content and particle size. For example, Gallagher and McDonald [16] evaluated the fiber size impact on the mechanical properties of WPCs. They used maple flour and high-density polyethylene. The results indicated that fiber size affects the modulus of elasticity (MOE) and the modulus of rupture (MOR). The results of these investigations showed that fiber content may increase tensile strength in WPCs. It is clearly shown that there is an ongoing interest to improve the tensile strength of WPCs using thermoplastics and sustainable materials. It is also important to mention that some of the polymers used were polypropylene (PP), polyethylene (PE), the chemical structures of which are shown in Figure 1.

**Figure 2:**
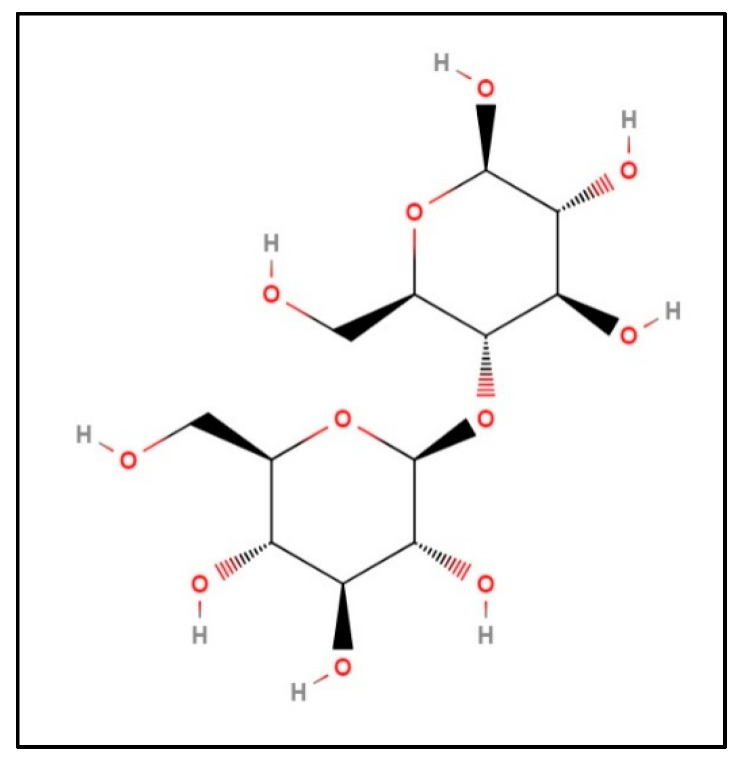
Cellolobiose [18].

These polymers have several advantages such as low cost, recyclable, high impact and good strength, among other properties. These characteristics become an import asset to the composites because they reinforce materials. Natural fibers, including from wood, have been used to impart good mechanical properties and to improve the sustainability of these plastics. Figure 2 shows the chemical structure of cellobiose, which is a component of wood [18]. Wood is made from three components composed of cellulose, hemicellulose and lignin [19]. Pecan wood is a hardwood with content consisting of 70% holocellulose, 21% lignin and 9% others [19].

As mentioned by Golofit et al. [20], composites from wood sources have been increasingly studied in the past ten years with the purpose of positively impacting the environment [21]. Within the development of WPCs, the analysis of wood degradation in the polymer matrix has been one focus of attention [22]. Understanding the other properties of WPCs is important since applications of the new developed material may be suitable for new applications where it is necessary to evaluate electrical or physical properties [23].

The mechanical properties of composites have been predicted using several micromechanical models. Various studies [11,15,24,25,26,27,28] have analyzed tensile properties based on experimental testing and theoretical modeling using models developed by Reuss, Vought, Hirsch, Halpin-Tsai, Modified Bowyer and Bader, among others, where the predicted values of the composites did not fit the test data very well. Little research has been performed on composites developed from tree pruning waste; specifically, reports from pecan trees are very limited. No study has been published that used pecan tree pruning flour as reinforcement in polyethylene. Some studies have reported on composites using pecan shells [8,12,14,22,23,29,30], although with different responses. In these studies, the composites were coupled with different types of coupling agents depending on the matrix type, most of which were polyolefin and polylactic acid. No studies have been reported on composites based on pecan wood flour or composites using pecan pruning waste. Due to the lack of information on the mechanical properties of high-density polyethylene (HDPE) and pecan wood flour composites, there is a need for assessing the mechanical properties of such material for potential application in different uses in construction or automotive. In this article, we developed and tested WPCs specimens based on pecan wood flour from tree pruning waste as reinforcement for HDPE and a coupling agent to enhance the bond between them. WPC specimens were fabricated using different weight ratios and particle sizes to determine their effect on the resulting mechanical properties. Statistical analysis was conducted to understand interaction effects on the mechanical response. Additionally, a micromechanical model was developed to better predict the tensile strength based on the shear interfacial strength.

## 2. Materials and Methods

### 2.1. Materials

ExxonMobil HDPE HD 6733 (ExxonMobil, Spring, TX, USA) was used as a matrix. This HDPE has good mechanical properties with a density of 0.950 g/cm^3^, melt index of 33/10 min at 190 °C (ASTM D1238). The coupling agent used in the composite was Polybond 3009 (Addivant, Danbury, CT, USA), which is a maleic anhydride modified high density polyethylene with a maleic anhydride content of 0.8 to 1.2%.

Pecan pruning waste was obtained from orchards in the region of Las Cruces, NM, USA. The pecan pruning waste was shredded as a first step, and then milled in a commercial hammer mill (Model 250E10-5780-12, Meadows Mill Inc., North Wikesboro, North Carolina, USA). Flour was milled using a ¼-inch screen as the initial step. The pecan wood flour was milled and sieved at 10, 20, 40 and 60 mesh. The flour from pecan wood was processed using a commercial sieve shaker.

### 2.2. Specimen Fabrication

In this study, specimens were fabricated in three steps. As a first step, pecan flour was sieved with 10-, 20-, 40- and 60-mesh screens (2000, 841, 400 and 250 microns) and dried for 24 h at 90 °C in an oven. In a second step, the composite blends were mixed in a twin extruder extrusion machine of 15 cc twin co-rotating screws, Xplore model DSM 15 cc capacity (DSM Research, Sittard, The Netherlands).

**Figure 3:**
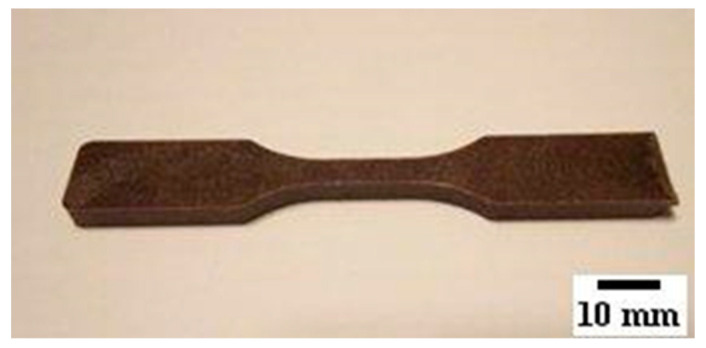
ASTM D638-14 Type V.

The extruder temperatures were set to 180 °C in all temperature zones; motor speed was set to 50 rpm with a maximum force of 8500 N; acceleration speed was set to 1000 rpm/min. Once the highest value of force was reached, a valve was opened to fill the injection molding cylinder. The third step was the injection molding process using a machine Xplore DSM 12 cc heating chamber, model Micro 12 cc (DSM Research, Sittard, The Netherlands). The injection molding machine was set with the mold temperature at 45 °C; the molding temperature was set at 190 °C; the three-stage injection process was set with the first stage at 9 bars for 5 s, the second stage at 11 bars for 5 s, and the third stage at 11 bars for 5 s. The coupling agent (CA) concentration was set between 3 and 5% [11,15]. Our preliminary testing results found that the value of 3.5% gave good results in the tensile response. Composites with pecan flour contents of 10, 30, 40, 50 and 60% were created, and a total of 6 type-V (ASTM D638-14 ASTM International, West Conshohocken, PA, United States.) specimens per test condition were injection molded (see Figure 3).

### 2.3. Mechanical Testing

A tensile test was performed using a universal tester Instron machine, model 5882. This machine has a built-in software called Blu hill used for data collection in the CSV format. The procedure was based on the ASTM D638-14 standard method to test the tensile strength of reinforced and unreinforced polymers using the type-V specimen. The head speed was set up at 1 mm/min. The modulus of elasticity was determined from the slope of the linear portion of the stress–strain plot.

### 2.4. Analysis of Variance

Testing data were analyzed to determine statistical effect of the factors mentioned for the pecan waste composite. A two-way analysis of variance (ANOVA) was used in order to determine the effect of each factor and their interactions. Minitab software was used for the analysis. The following results section provides more details about the resulting ANOVA.

## 3. Results and Discussion

Injection molded tensile samples were produced using HDPE and various concentrations of pecan wood particles. Experiments were conducted with specimens made of four mesh sizes, 10, 20, 40 and 60, and five weight contents, 10, 30, 40, 50 and 60%, which gives a total of 20 experimental runs. Polybond 3009 at a 3.5% weight was used as a coupling agent. Table 1 shows all the runs performed.

### 3.1. Tensile Properties

#### 3.1.1. Tensile Strength

Pecan branches were shredded and milled with bark of 16% of the wood weight [31]. The reinforcement gave a 16 to 44% average increase in the tensile strength of the matrix. A similar tensile response was reported by Adhikary et al. [15], as the pecan wood flour acted more as a reinforcement than a filler due to the effect of Polybond 3009 as coupling among pecan flour and HDPE. In their study, they used pinewood with ratio of 60% and found tensile results lower than the results in the present article. Furthermore, the study did describe that the composite had the highest tensile at a 40–50% ratio, whereas, in our study, higher values were at 50 and 60%. In another study by Facca et al. [24], they conducted experiments with a wood weight ratio of up to 60%. However, the tensile strength data reported do not agree with the tensile strength data of this study, as their values are lower. Table 2 displays the results of the analysis of variance (ANOVA) for tensile tests in all the pecan runs. Data of results in Appendix A.

**Figure 4:**
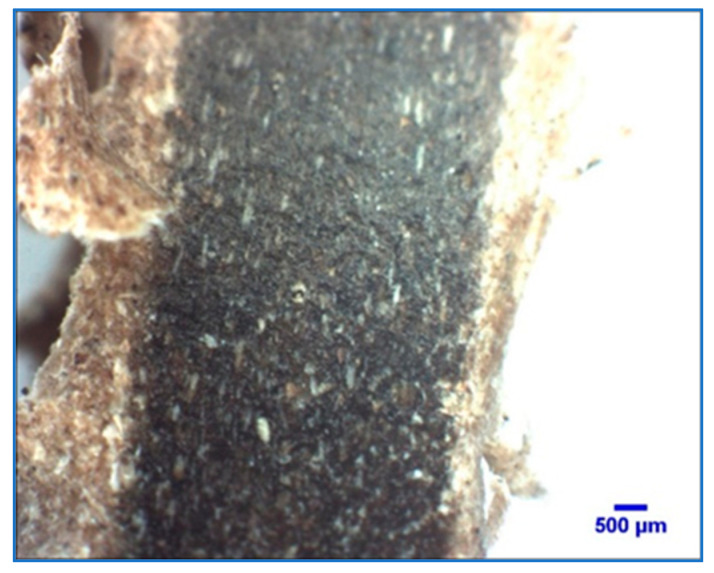
Machined sample of 60 Mesh (40% weight) shows composite mix.

Minitab^®^ 21.1 software was used to conduct the analysis of variance and to create Table 2. A significance level *p*-value was compared to an α-value = 0.05. Therefore, Table 2 shows that the *p*-value is less than the significance level for factors such as mesh size, weight fraction and the combination of mesh size and weight, and it is concluded that the mesh size and weight fraction have a significant effect on the response and tensile strength properties. Moreover, analyzing each *p*-value, the weight fraction of pecan has a major effect on the tensile properties. Figure 4 complements the above statement where a machined composite sample specimen with a 60-mesh size and 40% pecan wood weight fraction shows a good mix between HDPE and pecan wood.

**Figure 5:**
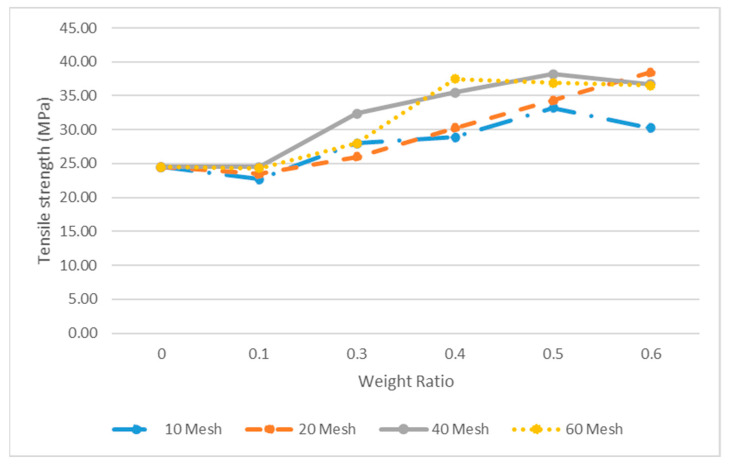
Tensile strength of pecan based on weight ratio and mesh size.

These results are in good agreement with those by Stark and Berger [32] and others in which tensile strength increases with an increasing wood weight ratio. Table 3 shows the results of the tensile strength of 40-mesh pecan flour at different weight ratios and a coupling agent content of 3.5%. Table 4 lists the average tensile strength values of all the mesh sizes by weight ratio and the equivalent volume fraction values that were estimated based on the density of HDPE and pecan wood. The tensile strength of the composites was increased by as much as 45%. The increase in tensile strength observed is dependent mainly on the pecan wood weight ratio. On average, the tensile values reached the maximum value at a 0.5 ratio and then decreased at a 0.6 ratio. The data related to tensile strength are shown in Figure 5 where the strength of the pecan composite increases as the weight increases until 50%. This result agrees with other studies from [11,15], where the maximum tensile strength reaches 50%. Other studies [24,26,27,32,33] observed the maximum tensile strength at 40% weight; however, Facca et al. [24] observed maximum values at 40% wood content for 40-mesh hardwood with HDPE. Furthermore, the stress values reported are lower than those reported in this article.

In addition, as observed in Figure 5, there is some variability in the tensile strength with respect to the mesh size, especially for the weight ratio at 50% and higher. For high weight ratios, WPCs produced from 10 mesh pecan flour were observed to have the lowest tensile strength; the poor bonding seems to be due to the volumetric particle size.

#### 3.1.2. Elastic Modulus

**Figure 6:**
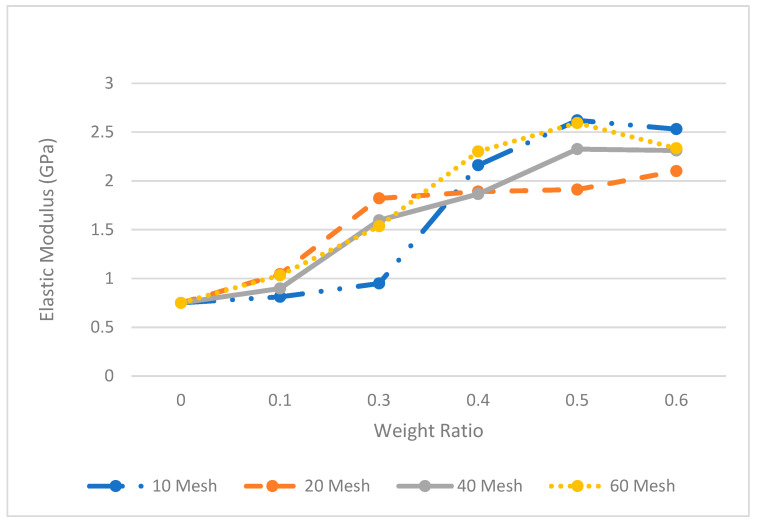
Modulus of Elasticity of Pecan composite.

The tensile modulus of elasticity (MOE) was estimated using the stress–strain relationship from the tensile test data. An analysis of variance was performed for MOE and is shown in Table 5. The effect of mesh size is not statistically significant, whereas the effect of weight ratio is quite significant (small *p*-value). Figure 6 and Table 6 show a significant increase in the MOE with respect to the pecan loading levels. The MOE values for all the mesh sizes with the same weight ratio showed an increase of 26 to 209%. This is highly significant, as other studies [33,34] reported an increase of up to 100%. The results from the MOE show inconsistent variability with respect to each mesh size.

The MOE experimental results show a similar behavior between tensile stress and weight ratio. The MOE increases as the weight increases, as described above. The incorporation of the pecan flour particles in the matrix improved the mechanical performance of the composite. Composites at high weight fractions of pecan flour were observed to have increased viscosity and volatiles, making the molding process more complex, requiring adjustments to the processing conditions.

### 3.2. Micromechanical Analysis

Several models have been developed to predict the mechanical properties of a composite. Some of the models proposed (e.g., [35,36]) do not consider the effect of the reinforcement particles shape and size in evaluating the composite properties shown in the following equation:

(1) $$\sigma_{c}=c1\sigma_{1}+c2\sigma_{2}$$

where *σ_c_* is the composite tensile strength, *σ*_1_ is the fiber tensile strength, *σ*_2_ is the matrix tensile strength and *c*_1_ and *c*_2_ are the volume fractions of fiber and matrix. This equation is known as the Voight equation, also called “the series model” [25]. A similar model was also proposed by the following Reuss equation. Fibers are parallel to the stress direction in this model.

(2) $$\sigma_{c}=\frac{\sigma_{m}*\sigma_{f}}{\sigma_{m}Vf+\sigma_{f}Vm}$$

where *σ_c_* is the composite tensile strength, *σ_f_* is the fiber tensile strength, *σ_m_* is the matrix tensile strength and *V_f_* and *V_m_* are the volume fractions of fiber and matrix. Other models consider factors such as fiber geometry, distribution and loading conditions to estimate the mechanical properties of a composite. These are sown in the Halpin-Tsai Equations (3) and (4), which provide a value of E and tensile in a composite with discontinuous fibers.

(3) $$\sigma_{c}=\sigma_{c}\left(1+\frac{AnVf}{1-nVf}\right)$$

(4) $$n\;=\frac{\left(\frac{\sigma_{f}}{\sigma m}\right)+1}{\left(\frac{\sigma f}{\sigma m}\right)+A}$$

In Equation (3), *σ_c_* is the composite tensile strength, *σ_f_* is the fiber tensile strength, *σ_m_* is the matrix tensile strength, *V_f_* and *V_m_* are the volume fractions of fiber and matrix, *n* is a factor for fiber orientation, and *A* is determined from the Einstein coefficient *K*. The Hirsch model in equation 5 considers a correcting factor in a model that combines series and parallel models. The correcting factor *x* is based on the fiber alignment, which is 0.4 for longitudinal and 0.1 for randomly oriented fibers [19].

(5) $$\sigma_{c}=x\left(\sigma mVm+\sigma fVf\right)+\left(1-x\right)\left(\frac{\sigma m\sigma f}{\sigma mVf+\sigma fVm}\right)$$

**Figure 7:**
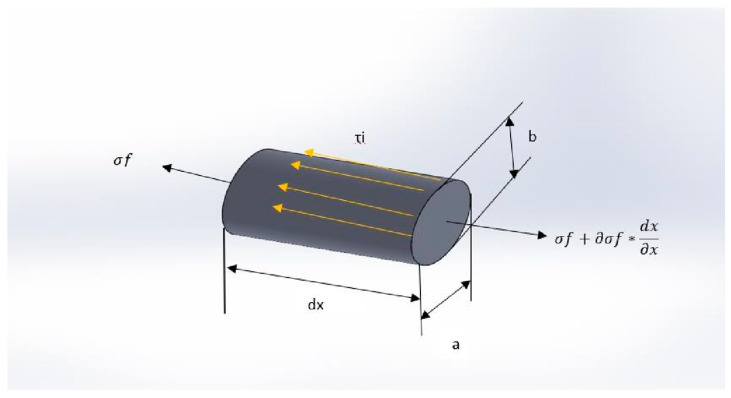
Stress elements in a fiber with ellipse element.

The preliminary results of the experimental data indicate that there is a significant effect of fiber size on mechanical properties. In this article, we used a modified micro mechanical model from Rosler, Harders and Baeker [37]. Figure 7 depicts the micromechanical model used in this study. In Figure 7, a fiber element *dx* with an ellipse shape is proposed, the tensile stress σf acts along the *x* axis and shear stress τi in the surface; this analysis was used to determine a correction factor for shape and size on fiber stress.

The correction factor for fiber normal stress was determined from the differential equation of stress equilibrium shown in Figure 7 and in the stress fiber Equation (6). In this equation, *σ_f_* is the tensile stress of fiber and τi is the interfacial shear stress of fiber, *a* is the major axis and *b* is the minor axis of fiber, and l is the fiber length. Combining this factor and using the equation from [24], a micromechanical model was developed considering elliptical fibers.

(6) $$\sigma_{f}=\tau i*\frac{\sqrt[{}]{\frac{1}{2}*a^{2}+b^{2}}}{a*b}*l$$

**Figure 8:**
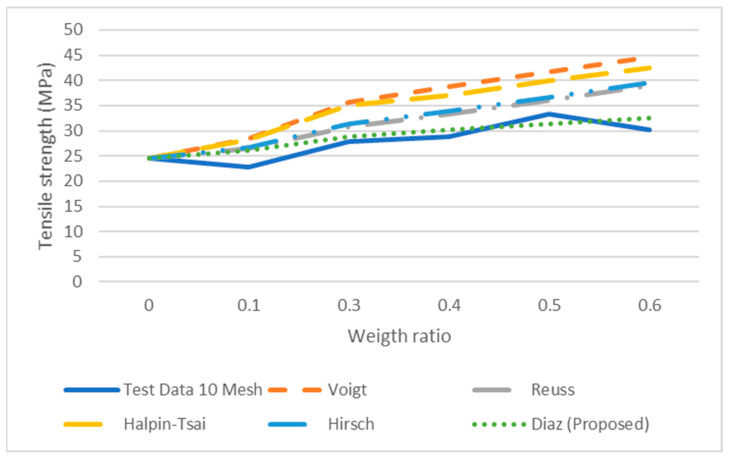
Micromechanical Tensile data with proposed model for 10 mesh.

**Figure 9:**
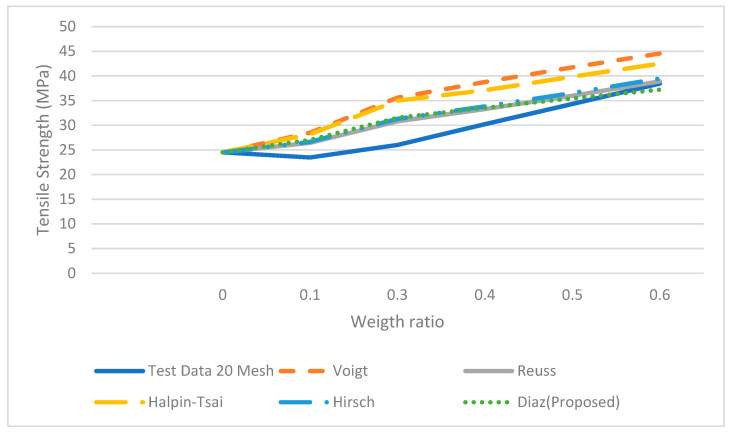
Micromechanical Tensile data with proposed model for 20 Mesh.

**Figure 10:**
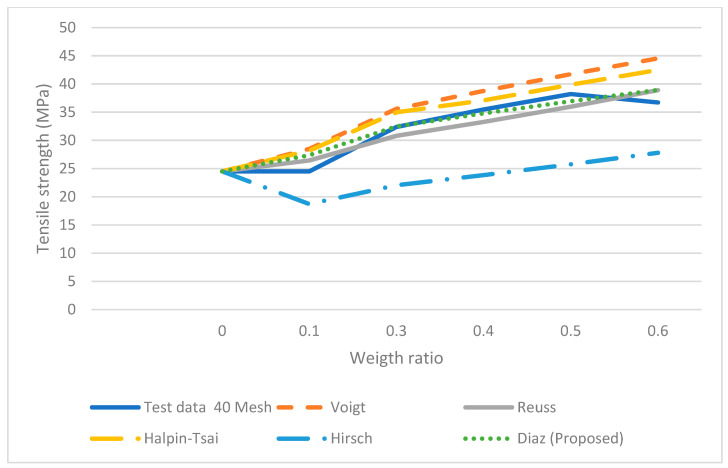
Micromechanical Tensile data with proposed model for 40 Mesh.

**Figure 11:**
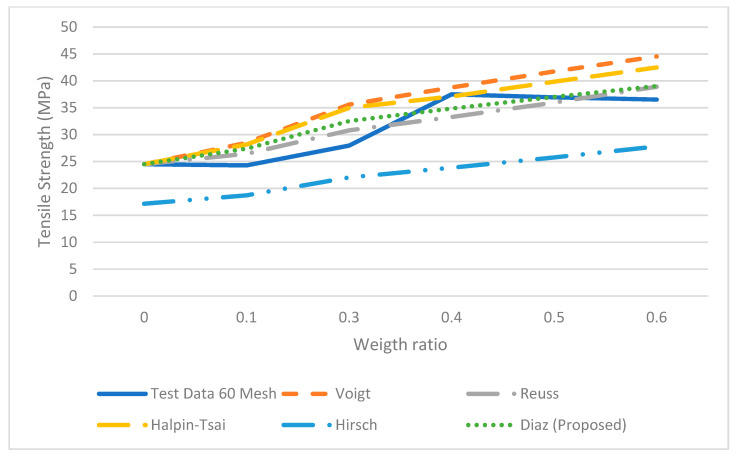
Micromechanical Tensile data with proposed model for 60 Mesh.

The equation proposed to calculate the tensile stress in a composite containing fibers with ellipse cross-sections is the following:

(7) $$\sigma_{c}=\tau Vf\left(\sqrt{\frac{1}{2}*\left(a^{2}+b^{2}\right)}\right)*\frac{l}{a*b}+\sigma m\left(1-Vf\right)\;$$

where σ_*c*_, τ, σ*m*, *Vf*, *l*, *a* and *b* are the tensile of the composite, the shear stress of the fiber, the tensile of the matrix, volume fraction of fiber, the length of the fiber and the major axis of the ellipse and the minor axis, respectively. This equation is the series or Voight equation modified by using a correction factor, which will adjust the response based on the elliptical shape while varying the length of the fiber. The parameters *a* and *b* in the equation represent a theoretical size of each particle and these approximate values were used to fit the mesh size. The tensile stresses were estimated using Equation (7). The results predicted by Equation (7) were more accurate than those obtained by the Vought, Reuss, Halpin-Tsai and Hirsch equations (see Figure 8, Figure 9, Figure 10 and Figure 11) for 10, 20 40 and 60 meshes.

Furthermore, the results from Vought, Reuss, Halpin-Tsai and Hirsch have a minimum difference in all the experimental runs since there is no correction factor for the fiber size and shape; the predicted results from the proposed micromechanical model are more consistent with the experimental data. Each set of experimental data with different mesh sizes shows non-monotonic behavior.

It was observed from three of four data sets that tensile strength increases as the weight ratio increases; then it decreases at the 50% weight ratio. The micromechanical models do not follow a similar trend in the experiment, i.e., the strength decreases at the 50% weight ratio. Moreover, as is observed, the models such as Halpin-Tsai or Hirsch do not fit the experimental data very well in all the weight ratios. The proposed micromechanical model, however, fits well in general and approximates the data near the 50% and 60% weight ratios.

## 4. Conclusions

Wood plastic composites were successfully produced from HDPE and milled pecan pruning waste flour. The tensile strength of the sample specimens obtained from this process were up to 46% higher than that of the HDPE matrix. The WPCs were fabricated using a 10 to 60% weight ratio of pecan flour and a coupling agent at 3.5%. The mesh sizes analyzed were 10, 20, 40 and 60 mesh. On each of the mesh sizes, the tensile strength was observed to increase with an increase in weight. These results are in agreement with those of other studies that use different waste sources. The data presented in this study clearly demonstrate that the WPCs produced with pecan pruning waste possess good mechanical properties. The Modulus of Elasticity values increased up to 200% compared to the matrix base value. The WPCs analyzed show that pecan wood acted not just as a filler, but also as a reinforcement; thus, the mechanical behavior was significantly improved.

A micromechanical model was proposed for predicting the mechanical properties of wood flour and fiber-reinforced composites. Some micromechanical models by Halpin Tsai, Voight, Reuss and Hirsch were also used to estimate tensile strength, and the difference in accuracy between these models and the proposed model was studied in comparison with the experimental data. The micromechanical model proposed uses the correction factor for elliptical fibers of varied sizes and shapes and provided a good fit to experimental the data obtained from the composite in all mesh sizes.

The tensile strength results for the WPCs material using pruned waste from pecan orchards indicates that it can be suitable for various applications that need to be further studied, such as in construction materials or interior automotive panels. Further efforts will be made to better asses the suitability of the WPC composite material in diverse applications and environments.

## Figures and Tables

**Table 1:** Wood plastic composite experimental test runs (Weight percent).

Composite Code	Mesh	Hdpe	Pecan Wood	Polybond 3009
HDPE	0	100	0	0
HDPE90P10	10	86.5	10	3.5
HDPE70P30	10	66.5	30	3.5
HDPE60P40	10	56.5	40	3.5
HDPE50P50	10	46.5	50	3.5
HDPE40P60	10	36.5	60	3.5
HDPE90P10	20	86.5	10	3.5
HDPE70P30	20	66.5	30	3.5
HDPE60P40	20	56.5	40	3.5
HDPE50P50	20	46.5	50	3.5
HDPE40P60	20	36.5	60	3.5
HDPE90P10	40	86.5	10	3.5
HDPE70P30	40	66.5	30	3.5
HDPE60P40	40	56.5	40	3.5
HDPE50P50	40	46.5	50	3.5
HDPE40P60	40	36.5	60	3.5
HDPE90P10	60	86.5	10	3.5
HDPE70P30	60	66.5	30	3.5
HDPE60P40	60	56.5	40	3.5
HDPE50P50	60	46.5	50	3.5
HDPE40P60	60	36.5	60	3.5

**Table 2:** Resulted Analysis of Variance (ANOVA) analysis of tensile strength of Pecan wood plastic Composites (WPCs) from Minitab^®^ 21.1 Software.

Source of Variation	Degrees of Freedom	Seq Sum of Squares	Contribution	Adjusted Sums of Squares	Adjusted Mean Squares	Test Statistic F-Value for the Model	Significance Level *p*-Value
Mesh size	3	433.3	11.39%	433.3	144.448	30.28	5.18 × 10^−16^
Weight fraction	4	2557.6	67.20%	2557.6	639.395	134.04	1.1 × 10^−18^
Mesh size * Weight	12	338.0	8.88%	338.0	28.166	5.90	1.15 × 10^−7^
Error	100	477.0	12.53%	477.0	4.770		
Total	119	3805.9	100.00%				

* The asterisk meaning is interaction effect between the two factors.

**Table 3:** Average tensile strength of pecan WPCs for 40 mesh.

Pecan Weight Ratio	Volume Fraction	Tensile (MPa)	Std Deviation	Tensile% Increase
0	0	24.50	0	0
0.1	0.134	24.503	0.893	0.010
0.3	0.374	32.380	1.337	32.160
0.4	0.482	35.480	1.466	44.820
0.5	0.582	38.220	2.950	56.000
0.6	0.676	36.710	2.990	49.840

**Table 4:** Tensile strength average values for all mesh sizes.

Weight	Volume Fraction	Tensile (MPa)	Tensile% Increase
0	0	24.500	0
0.1	0.134	24.505	0.0
0.3	0.374	28.590	16.7
0.4	0.482	33.050	34.9
0.5	0.582	35.670	45.5
0.6	0.676	35.440	44.5

**Table 5:** ANOVA analysis of Elastic Modulus of pecan WPCs.

Source of Variation	SS	DF	MS	F	*p*-Value	F Crit
Mesh Size	0.11899	3	0.039665	0.537574	0.665421	3.490295
Weight Ratio	5.91306	4	1.478267	20.03492	3.03 × 10^−5^	3.259167
Error	0.88541	12	0.073785			
Total	6.91747	19				

**Table 6:** Elastic modulus results for pecan wood composite (average).

Weight	Volume Fraction	Elastic Modulus (E) (GPa)	Standard Deviation	E% Increase
0	0	0.75	0	0
0.1	0.1343	0.946	0.096	26.2
0.3	0.3745	*1.475*	0.321	96.7
0.4	0.4822	*2.054*	0.183	173.8
0.5	0.5828	*2.362*	0.284	214.9
0.6	0.6769	*2.318*	0.152	209.1

## Data Availability

The data used in this study are within the article.

## Appendix A. Data of the Study from Excel Format

**Table:**

**Pecan Waste Stress Data**		
**Mesh**	**Weight(%)**	**Tensile (MPa)**
10	10	21.278
10	10	21.499
10	10	22.735
10	10	20.523
10	10	23.982
10	10	24.616
10	30	32.119
10	30	27.018
10	30	29.187
10	30	25.849
10	30	25.986
10	30	26.761
10	40	26.097
10	40	28.236
10	40	27.889
10	40	31.306
10	40	31.196
10	40	28.624
10	50	33.003
10	50	33.940
10	50	33.527
10	50	34.417
10	50	33.259
10	50	33.380
10	60	22.703
10	60	27.681
10	60	34.677
10	60	27.512
10	60	35.691
10	60	33.429
20	10	23.687
20	10	21.485
20	10	23.083
20	10	24.590
20	10	23.142
20	10	24.292
20	30	26.173
20	30	25.883
20	30	27.679
20	30	26.054
20	30	24.199
20	30	26.076
20	40	29.720
20	40	30.501
20	40	29.349
20	40	30.977
20	40	30.103
20	40	30.696
20	50	31.618
20	50	37.457
20	50	31.536
20	50	35.139
20	50	36.394
20	50	33.753
20	60	37.505
20	60	34.012
20	60	40.334
20	60	40.303
20	60	40.657
20	60	37.903
40	10	22.865
40	10	24.936
40	10	25.435
40	10	24.640
40	10	24.236
40	10	24.908
40	30	32.880
40	30	31.346
40	30	32.696
40	30	33.782
40	30	30.238
40	30	33.369
40	40	36.338
40	40	34.491
40	40	36.383
40	40	37.522
40	40	34.345
40	40	33.819
40	50	40.644
40	50	40.156
40	50	36.398
40	50	40.570
40	50	33.350
40	50	38.242
40	60	34.302
40	60	38.961
40	60	34.907
40	60	38.401
40	60	40.588
40	60	33.118
60	10	23.665
60	10	24.488
60	10	24.174
60	10	23.863
60	10	24.411
60	10	25.130
60	30	31.768
60	30	27.668
60	30	28.188
60	30	27.362
60	30	29.310
60	30	23.615
60	40	39.040
60	40	36.378
60	40	38.624
60	40	37.550
60	40	35.671
60	40	37.830
60	50	37.126
60	50	38.760
60	50	36.135
60	50	38.641
60	50	34.714
60	50	36.205
60	60	35.448
60	60	35.381
60	60	39.456
60	60	34.553
60	60	40.417
60	60	33.938
